# iSTART-II: An Update on the i Support Therapy–Access to Rapid Treatment (iSTART) Approach for Patient-Centered Therapy in Mild-to-Moderate Ulcerative Colitis

**DOI:** 10.3390/jcm12031142

**Published:** 2023-02-01

**Authors:** Ferdinando D’Amico, Fernando Magro, Benedicte Caron, Axel Dignass, Vipul Jairath, Ailsa Hart, Paulo Gustavo Kotze, Kristine Paridaens, Sameer Al Awadhi, Taku Kobayashi, Britta Siegmund, Laurent Peyrin-Biroulet, Silvio Danese

**Affiliations:** 1 Gastroenterology and Endoscopy, IRCCS Ospedale San Raffaele and Vita-Salute San Raffaele University, 20132 Milan, Italy; 2 Department of Biomedical Sciences, Humanitas University, Pieve Emanuele, 20072 Milan, Italy; 3 Department of Gastroenterology, Centro Hospitalar São João, 4200-319 Porto, Portugal; 4 Department of Gastroenterology, CHRU-Nancy, University of Lorraine, 54000 Nancy, France; 5 Inserm, NGERE, University of Lorraine, 54000 Nancy, France; 6 Department of Medicine I, Agaplesion Markus Hospital, Goethe-University, 60323 Frankfurt am Main, Germany; 7 Department of Medicine, Division of Gastroenterology, Western University, London, ON N6A 3K7, Canada; 8 St Mark’s Hospital, Middlesex, Harrow HA1 3UJ, UK; 9 Colorectal Surgery Unit, Hospital Universitário Cajuru, Pontificia Universidade Católica do Paraná (PUCPR), Curitiba 80215-901, Brazil; 10 Ferring Pharmaceuticals, 1162 Saint-Prex, Switzerland; 11 Digestive Diseases Unit, Rashid Hospital, Dubai 003206, United Arab Emirates; 12 Center for Advanced IBD Research and Treatment, Kitasato University Kitasato Institute Hospital, Tokyo 108-8642, Japan; 13 Medizinische Klinik m. S. Gastroenterologie, Infektiologie und Rheumatologie, Charité-Universitätsmedizin Berlin, Corporate Member of Freie Universität Berlin, Humboldt-Universität zu Berlin, and Berlin Institute of Health, 10178 Berlin, Germany

**Keywords:** ulcerative colitis, inflammatory bowel disease, mild disease, fecal calprotectin

## Abstract

The i Support Therapy–Access to Rapid Treatment (iSTART) was an initiative to improve patient-centered management in mild-to-moderate ulcerative colitis (UC). Our aim was to update the iSTART recommendations in order to include fecal calprotectin (FC) in the monitoring of patients with UC and improve their management. Twelve physicians from nine countries worldwide attended a virtual international consensus meeting on 4 May 2022. Data from three systematic reviews were analyzed, and a new systematic review investigating all studies reporting measurement of FC at home was conducted. Based on literature evidence, statements were formulated, discussed, and approved by voting. Statements were considered approved if at least 75% of participants agreed with a proposed statement. Fourteen statements were approved. Based on this consensus, FC measurement should be routinely performed for monitoring patients with mild-to-moderate UC to identify disease relapses early and initiate an appropriate treatment. Further studies are needed to assess whether self-monitoring of FC is associated with better disease control and improved patients’ quality of life.

## 1. Introduction

Growing evidence suggests that ulcerative colitis (UC), like Crohn’s disease (CD), is a progressive disease that can evolve into structural damage and be associated with narrowing and stenosis [1,2,3]. For this reason, it is legitimate to hypothesize that timely treatment may guarantee better symptom control, resulting not only in an improved quality of life but also in a better long-term prognosis. To date, mesalamine is the first-line therapy for UC patients with mild-to-moderate disease according to international guidelines [4,5]. In case of nonresponse or disease recurrence, the optimization of oral and topical mesalamine is the best option. If this strategy is not effective, the addition of a corticosteroid is recommended, and budesonide MMX should be preferred in subjects with mild-to-moderate disease activity [6]. The key to achieving disease control is accurate patient monitoring in order to identify active disease early and set up appropriate treatment. The i Support Therapy–Access to Rapid Treatment (iSTART) was an initiative to improve patient-centered management to UC [6]. Based on the recommendations of this expert consensus, patients had to be educated on how to monitor their disease activity (using validated patient reported outcomes) and self-optimize their treatment in case of clinical relapse. This approach was intended to avoid therapeutic delays, improving disease management and patients’ quality of life. This strategy could also allow for early identification of disease exacerbation and appropriate treatment while limiting disease progression. However, the monitoring of patients with inflammatory bowel diseases (IBD) has changed considerably in recent years, and the measurement of fecal calprotectin (FC) has obtained an increasingly central role. Indeed, FC helps to evaluate disease activity and response to therapy, predicts clinical relapses and endoscopic and histological activity, and is recognized as an intermediate treatment target [7,8,9]. Consequently, this consensus aimed to update the iSTART recommendations in order to include FC in the monitoring of patients with mild-to-moderate UC and improve their management.

## 2. Materials and Methods

The literature evidence on FC was obtained from three systematic reviews previously published by our group [7,8,9]. In addition, a new systematic review was conducted using Pubmed, Cochrane library, and Web of Science databases in order to investigate all studies reporting measurement of FC at home in patients with IBD. Details of the systematic review are provided in the supplementary Table S1 and supplementary Figure S1. Based on this evidence, 13 statements were created by 3 authors (FD, LPB, and SD) (Table 1). Then, a virtual international meeting was conducted on 4 May 2022. Twelve experts from nine countries worldwide (Italy, France, Germany, Portugal, United Kingdom, Canada, Japan, Brazil, and United Arab Emirates) participated in the meeting. First, the available literature data concerning FC in the management of patients with IBD were shown. Subsequently, each proposed statement was presented and voted by all participants in an anonymous manner using a digital polling system. Agreement was defined as at least 75% of participants voting for a proposed statement. If a 75% agreement was not achieved, it was further discussed, amended, and followed by a second round of voting. If agreement could not be reached after two rounds of voting, the statement was definitely excluded. Further statements could be proposed by the experts and subsequently discussed and voted on according to the methods described above. All experts were involved in drafting the manuscript and approved its final version.

## 3. Results

Three statements were approved after the first round and six statements after the second round of voting. Two statements were merged and then approved. Two statements were split into two parts each and approved after a second round of voting. Only one statement was dropped after two rounds of votes. The list of approved statements is provided in Table 2.

**Statement 1: Fecal calprotectin is a reliable biomarker for differentiating ulcerative colitis from functional disorders (agreement 100%).**

IBD and irritable bowel syndrome (IBS) share some symptoms such as diarrhea and abdominal pain. To date, there is no specific test for IBS, and the diagnosis of IBS is reached after excluding other diseases [10]. A systematic review and meta-analysis by Waugh and colleagues evaluated the accuracy of FC in distinguishing IBS and IBD [11]. A cut-off level of 50 µg/g was associated with a sensitivity of 93% and specificity of 94% in distinguishing between IBD and IBS in adults. Similarly, the same FC threshold provided a sensitivity varying from 83% to 100% and a specificity from 60% to 100% in the pediatric population, suggesting the reliability of this biomarker. The measurement of FC allows the screening of patients who are candidates for second-level procedures, such as colonoscopy, and reduces the number of unnecessary invasive procedures, ultimately leading to significant cost savings [12]. For this reason, FC measurement should always be recommended in the diagnostic workup of patients with suspected IBD or IBS.

**Statement 2: Fecal calprotectin is a reliable biomarker to evaluate disease activity in patients with ulcerative colitis (agreement 100%).**

Calprotectin is a protein contained in the cytoplasm of neutrophils. It is measured in the stools to assess the presence of gut inflammation, and it is associated with disease outcomes [13]. A study by Tibble and colleagues showed that an increase in FC concentrations was associated with a high risk of clinical recurrence in UC patients in clinical remission [14]. An FC value > 50 µg/g predicted clinical disease recurrence with a sensitivity and specificity of 90% and 83%, respectively. A systematic review and meta-analysis including 19 cohort and case-control studies evaluated the diagnostic accuracy of FC in patients with active IBD using endoscopy as gold standard [15]. Interestingly, FC had reliable sensitivity (0.88, 95% confidence interval (CI) 0.84–0.90) and specificity (0.73, 95% CI 0.66–0.79), resulting in more sensitive than C-reactive-protein (CRP) in evaluating disease activity. To date, there are several tests for measuring FC. The enzyme-linked immunosorbent assay (ELISA) tests and automated ELISA tests are the most frequently used tools, have an accurate analytical performance, and should be preferred [16]. In addition, point of care (POC) tests and home tests have been developed. They proved to be accurate and represent a valid alternative to traditional tests [16,17,18,19,20].

**Statement 3: Fecal calprotectin levels are correlated with endoscopic disease activity in patients with ulcerative colitis (agreement 100%).**

Colonoscopy is still the gold standard for evaluating UC patients and making treatment decisions. However, endoscopic examinations are invasive, expensive, and poorly tolerated by patients [21,22]. Currently, there is robust evidence regarding the correlation between endoscopic disease activity and FC values in UC. Walsh et al. evaluated the association between FC and endoscopic disease activity assessed by the Ulcerative Colitis Endoscopic Index of Severity (UCEIS) score [23]. A strong correlation was identified (r = 0.741, 95% CI 0.289–0.922, *p* < 0.003), and an FC cut-off of 170 µg/g predicted remission or endoscopic activity (UCEIS ≤ 1 and UCEIS ≥ 4, respectively). Similarly, Theede et al. showed that FC was associated with endoscopic activity measured by UCEIS and Mayo endoscopic score (MES) [24]. Of note, FC values < 192 µg/g predicted mucosal healing. A prospective observational study enrolling 228 patients with UC investigated the correlation between endoscopy and several noninvasive markers of inflammation (Lichtiger index, FC, CRP, platelets, hemoglobin, and blood leukocytes) [25]. FC was the most correlated marker with endoscopic activity (r = 0.821), and an FC cutoff of 57 µg/g had a sensitivity of 91% and a specificity of 90% to detect endoscopically active disease (modified Baron Index ≥ 2).

Although there are factors that can influence the concentration of FC and affect its dosage (e.g., intake of non-steroidal anti-inflammatory drugs and proton pump inhibitors, incorrect sampling or storage), FC is a reliable predictor of endoscopic activity and can be used to make therapeutic decisions by reducing the number of performed colonoscopies and allowing for rapid therapeutic choice.

**Statement 4: Fecal calprotectin levels are correlated with histological disease activity in patients with ulcerative colitis (agreement 92%).**

Up to 40% of UC patients in endoscopic remission exhibit histological disease [26]. This persistent microscopic activity is associated with an increased risk of relapse [27,28]. For this reason, histological activity is now recognized as an additional nonformal target and should be considered in the management of patients with UC [9]. Growing evidence demonstrates the association between FC and histology. A systematic review by D’Amico et al. investigated all studies reporting the correlation between FC and histology in UC patients [8]. Twelve studies enrolling over 1000 patients were included. Interestingly, an association between FC and histology was found in all studies. FC values ranging from 40.5 to 250 μg/g helped to distinguish between histological remission and microscopic disease activity. Moreover, a post hoc analysis from a phase 4 trial revealed that an FC cutoff between 75 and 100 μg/g predicted histological remission of disease in patients with mild-to-moderate UC [29].

**Statement 5: A fecal calprotectin value greater than 250 µg/g suggests the presence of active disease (agreement 100%).**

While there is common agreement on the reliability of FC as a predictor of outcomes, there is no globally accepted and validated cutoff. Indeed, robust evidence suggests that FC values below 100 μg/g are associated with disease remission, while values above 250 μg/g predict an active disease (after exclusion of infectious causes) [9]. In the range between 100 and 250 μg/g, there is a “gray area” of values that could be associated with different degrees of activity in UC, varying from histological remission to endoscopic remission (Mayo 0) and mild endoscopic activity (Mayo 1). Of note, Hart et al. found that a cutoff of 170 μg/g was able to distinguish patients with MES 0 from those with MES ≥ 1 (64% sensitivity, 74% specificity, AUC = 0.743 [95% CI 0.67–0.82], *p* < 0.001), while a cutoff of 135 μg/g allowed to identify patients with histological remission (defined as Geboes score < 3.1) [30]. In this “grey area” scenario, repeated FC measurement could be performed to rule out a false positive. Alternatively, other factors, such as patient symptoms or noninvasive monitoring techniques like small bowel ultrasound, could be considered.

**Statement 6: Fecal calprotectin is a reliable biomarker to evaluate the response to treatment in patients with ulcerative colitis (agreement 100%).**

To date, FC is increasingly used to assess response to therapy both in clinical practice and in randomized clinical trials. A post hoc analysis of a phase 3 trial investigated the effect of induction therapy on FC concentrations [31]. After 6 weeks of treatment, patients with clinical and endoscopic remission had lower levels of FC than those with persistent symptoms and ulcers on colonoscopy. FC < 150 μg/g was associated with clinical and endoscopic remission, thus supporting the role of FC as a surrogate biomarker. Similarly, an analysis of 3 cohorts of prospectively enrolled patients investigated the accuracy of FC in discriminating histological response and histological remission after treatment [32]. Patients who achieved histological response or histological remission (assessed by Geboes score (GS) or Robarts histopathology index [RHI]) had lower FC (µg/g) values than subjects with microscopic disease activity (GS: 73.00 vs. 525.00, *p* < 0.001; RHI: 73.50 vs. 510.00, *p* < 0.001 for histological response; GS: 76.00 vs. 228.00, *p* < 0.001; RHI: 73.50 vs. 467.00, *p* < 0.001 for histological remission).

**Statement 7: Fecal calprotectin is a reliable biomarker to monitor disease activity in patients with ulcerative colitis (agreement 100%).**

Endoscopic and histological activity guide therapeutic decisions in UC [9]. However, it is necessary to perform a colonoscopy or flexible sigmoidoscopy to obtain these data. The close correlation between FC and histology and endoscopy could limit the number of endoscopic procedures. FC is a valuable marker for monitoring disease activity and allows stratification of patients requiring invasive procedures. In those with an increase in FC levels (≥250 µg/g), endoscopic evaluation should be indicated, while in patients with normal FC values (<100 µg/g), no further testing should be performed except for endoscopic colorectal cancer surveillance.

**Statement 8: Persistent high values of fecal calprotectin predict clinical disease relapse in asymptomatic patients (agreement 100%).**

FC has an increasingly central role as a predictor of disease recurrence in UC patients. A prospective observational study by Guardiola et al., including patients with clinical and endoscopic remission, showed that patients with active histologic inflammation had a significantly higher median level of FC (278 μg/g) than those without active histologic inflammation (68 μg/g) (*p* = 0.002) [33]. Another observational study evaluated the accuracy of consecutive FC measurements to predict flares in IBD patients [34]. The included patients were in clinical remission of disease and were treated with stable therapy for at least 6 months. Patients were monitored clinically every 2 months, and FC was measured every 4 months with an overall follow up of 12 months. Approximately one third of patients experienced a relapse during the study (31.6%). Patients who relapsed had significantly higher FC concentrations than those who remained in remission (477 μg/g vs. 65 μg/g, *p* < 0.005). An FC value of 130 μg/g predicted remission, while an FC > 300 μg/g was associated with relapse.

**Statement 9: Fecal calprotectin should be a treatment target in patients with ulcerative colitis (agreement 92%).**

FC predicts endoscopic/histological activity and disease recurrence in UC. According to the new Selecting Therapeutic Targets in Inflammatory Bowel Disease (STRIDE) II recommendations, FC is an intermediate target for UC patients, and therapy should be optimized or changed if this target is not met [9]. A recent multinational, survey-based study confirmed the key role of FC in the management of patients with mild-to-moderate UC, emphasizing the need to optimize therapy based on FC values [35]. This strategy could allow for early disease treatment by maintaining remission, preventing clinical relapses, and improving patients’ quality of life [35].

**Statement 10: Fecal calprotectin should be measured before starting any therapy for ulcerative colitis (agreement 100%).**

The relevance of FC measurement depends not only on the absolute value of FC but is mainly associated with the trend of FC concentrations. A study by Molander et al. assessed whether FC measured after induction therapy predicted outcomes during the maintenance phase [36]. FC was normalized (≤100 μg/g) in approximately 50% of patients after induction. Importantly, most patients (84%) who had normal FC values after induction were in clinical remission after 12 months of follow up. On the other hand, only a small percentage (38%) of patients who had elevated FC concentrations (≥100 μg/g) after induction were in remission at 12 months. A post-induction FC concentration of 139 μg/g was identified as a predictor of clinical disease recurrence within one year (sensitivity 72% and specificity 80%). For this reason, it is essential to have a baseline FC value before starting any treatment in order to monitor FC concentrations and predict any recurrences over time.

**Statement 11: Fecal calprotectin should be measured before optimizing any therapy for ulcerative colitis (agreement 100%).**

Measuring the FC before optimizing the therapy allows for monitoring the response to treatment and possibly anticipating endoscopic evaluation or changing the therapy in case of failure. Interestingly, a prospective study evaluated the role of FC in UC patients in clinical remission before de-escalating therapy [37]. FC levels were inversely associated with time to relapse [*p* < 0.0001]. An FC value > 100 µg/g predicted a worsening of symptoms within one year of therapeutic de-escalation (AUC = 0.84). Only one patient with FC < 100 µg/g experienced clinical relapse within 3 months, while most patients with FC > 100 µg/g (85%) had a relapse during the follow-up. Based on these data, FC should be measured before each therapeutic change (including both treatment escalation and de-escalation) in order to assess not only the response to therapy but also to predict any relapse.

**Statement 12: Fecal calprotectin should be measured at the end of induction therapy, during maintenance, or in case of clinical exacerbation of disease (agreement 100%).**

FC measurement along with clinical evaluation should be the first-level assessment of all patients with mild-to-moderate UC. FC should always be measured at the end of induction therapy to assess therapeutic response and predict the risk of nonresponse. A post hoc analysis of the GEMINI 1/GEMINI LTS and VARSITY trials assessed accuracy of post-induction FC in identifying activity of disease [38]. An FC value < 250 µg/g vs. FC > 250 µg/g was associated with a higher probability of achieving clinical remission (odds ratio [OR], 4.03; 95% CI, 2.78–5.85), endoscopic remission (OR, 4.26; 95% CI, 2.83–6.40), and histologic remission (OR, 5.54; 95% CI, 3.77–8.14 based on the Robarts Histopathology Index; OR, 6.42; 95% CI, 4.02–10.26 based on the Geboes score) at week 52 and a lower risk of surgery (hazard ratio, 0.296; 95% CI, 0.130–0.677) and UC-related hospitalization (hazard ratio, 0.583; 95% CI, 0.389–0.874). A retrospective cohort study enrolling patients with Crohn’s disease investigated the role of FC before and after treatment in predicting primary nonresponse to drugs [39]. All enrolled patients had elevated FC values prior to treatment (median: 860 µg/g). After induction, approximately two thirds of patients achieved a response with a significant reduction in FC values (*p* < 0.0001). Patients who had less than 70% reduction in FC between pre- and post-treatment had the highest prognostic value for nonresponse with 99% sensitivity and 96% specificity. Of note, FC monitoring is also important during maintenance of disease remission. Tight monitoring with FC measurement every three months proved to be an effective strategy in identifying early relapses and optimally treating patients [40,41]. At the same time, the assessment of FC in case of clinical relapse should be recommended in order to confirm the presence of inflammation and support the need for medical therapy escalation.

**Statement 13: Escalation therapy should be performed in asymptomatic patients in case of persistent elevated fecal calprotectin values (agreement 83%).**

Certain medication (e.g., non-steroidal anti-inflammatory drugs and proton pump inhibitors), lifestyle (obesity and physical inactivity), concomitant disorders (e.g., diverticulosis and irritable bowel symptoms), or errors in stool sample storage can affect the FC dosage [16]. Consequently, if an increase in FC is detected and it is not justified by the symptoms and considered suspicious for a false positive by the clinician, the FC measurement should be repeated. The persistent increase in FC after a serial measurement confirms the presence of inflammation and is associated with a high risk of relapse and requires treatment [9]. A randomized controlled trial by Osterman and colleagues evaluated whether mesalamine optimization (>2.4 g day) was associated with a reduction of FC < 50 μg/g in patients with UC in clinical remission but with increased FC [42]. Significantly more patients undergoing therapy optimization achieved normalization of FC than the control group (26.9% vs. 3.8%, *p* = 0.0496). Moreover, patients with FC levels < 200 μg/g experienced fewer relapses than those with FC > 200 μg/g (*p* = 0.01).

**Statement 14: Home measurement of fecal calprotectin is a useful and reliable method for monitoring disease activity in patients with ulcerative colitis (agreement 100%).**

The development of home tests for FC monitoring is a useful innovation in the management of patients with IBD [19,20,43,44,45,46,47,48,49,50,51,52,53,54,55,56,57,58,59,60,61]. To date, several devices are available. They help avoid problems related to the storage and transport of the stool sample, but their use requires adequate training and high compliance by patients. Home tests provide a semiquantitative FC value and are highly correlated with traditional ELISA tests for FC concentrations ≤ 500 μg/g, thus resulting in reliable alternatives for measuring FC [43,44]. Patients could monitor their FC whenever they perceive a worsening of symptoms. The FC home dosage could allow early identification of any relapses and early treatment, reducing the onset of symptoms and positively impacting the quality of life of patients.

## 4. Discussion

**Figure 1:**
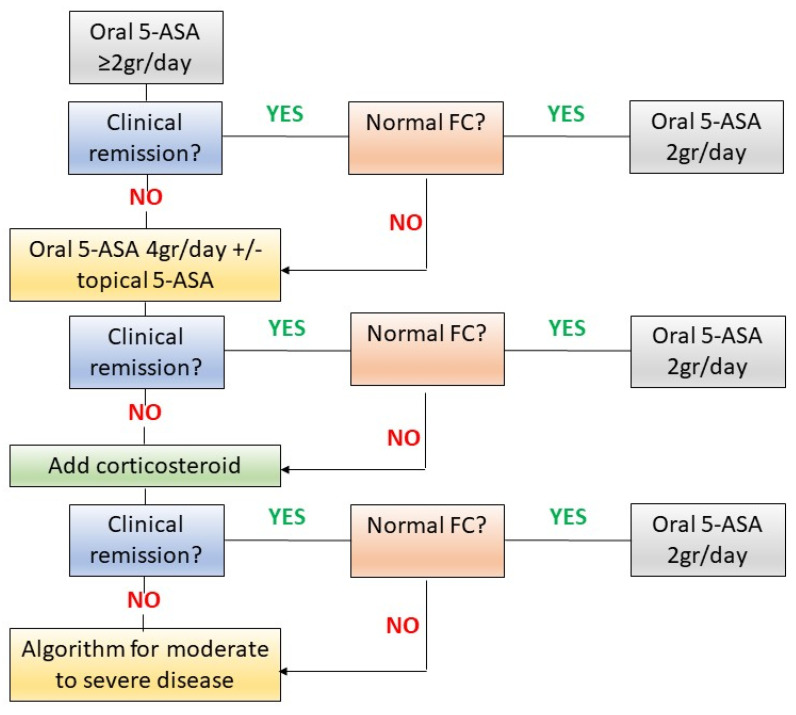
Proposed algorithm for the management of patients with mild-to-moderate ulcerative colitis. FC: fecal calprotectina. 5-ASA: mesalamine.

The proposed algorithm is summarized in Figure 1. Oral and topical mesalamine should be the first-line therapy for patients with mild-to-moderate UC according to international guidelines [4,5,62]. In mild UC, oral mesalamine at a dosage of ≥2 g/day is recommended to induce remission followed by a mesalamine 2 g/day for the maintenance phase. In patients with moderate disease, mesalamine at a dosage of 4 g/days should be recommended and combined with 1 g mesalamine suppositories (if proctitis) or 4 g mesalamine enemas (if left-sided colitis) [63]. If clinical remission is not achieved or high FC values persist after therapy with mesalamine 2 g/day, treatment optimization with oral mesalazine 4 g/day and topical mesalamine is recommended. In patients who do not achieve a clinical and biochemical response, second-line therapy should be considered. Corticosteroids are the recommended drugs in such a scenario. They should be used for the shortest possible duration and at the lowest effective dosage. For patients with moderate clinical symptoms and no systemic signs (e.g., high fever, severe anemia or tachycardia and hypotension), second generation corticosteroids, such as budesonide MMX, should be preferred as an add-on due to their safety [6]. On the other hand, in patients with severe disease activity, traditional corticosteroids, such as prednisone and methylprednisolone, should be used. FC measurement should be performed whenever there is a re-exacerbation of symptoms and before any therapeutic change. Furthermore, FC should be measured at the end of the treatment induction phase, and if remission is achieved, it should be monitored every 3–4 months during the maintenance phase. Although FC is a very useful tool in clinical practice to guide therapeutic decisions, it is important to underline that several factors can affect its result. In fact, errors in the sampling or storage of the faeces or the presence of liquid or bloody faeces can cause an alteration of FC values [16]. Furthermore, drugs, concomitant diseases (e.g., colorectal neoplasia, infections, diverticula, irritable bowel syndrome, and cirrhosis), the presence of pseudopolyps, and lifestyle habits (e.g., obesity and physical inactivity) can modify the levels of FC [16]. For this reason, all these aspects should be considered when interpreting FC results, and in doubtful cases, FC repetition or the endoscopic assessment should be considered.

## 5. Conclusions

Mild-to-moderate ulcerative colitis negatively impacts patients’ quality of life and requires adequate monitoring and treatment. Symptom control and fecal calprotectin have a central role in the management of patients with ulcerative colitis and should always be considered in the decision-making process in order to favor a self-monitoring of disease activity, avoid invasive and unnecessary endoscopic procedures, and ensure disease control. Patients should be sensitized and carefully informed about the relevance of clinical and biochemical data in order to early identify disease relapses and set up an equally early treatment. An ongoing randomized clinical trial (NCT04340895) will provide further evidence clarifying whether an approach based on symptom assessment and fecal calprotectin measurement is associated with greater benefits in terms of disease control and quality of life compared with the evaluation of symptoms alone in patients with mild-to-moderate ulcerative colitis.

## Figures and Tables

**Table 1:** Proposed statements regarding the measurement of fecal calprotectin in the management of patients with inflammatory bowel diseases.

		Agreement after the First Round of Voting
Statement 1	Fecal calprotectin is a reliable biomarker for differentiating ulcerative colitis from functional disorders.	100%
Statement 2	Measurement of fecal calprotectin is cost-effective in identifying patients suspected of ulcerative colitis.	<75% *
Statement 3	Fecal calprotectin is a reliable biomarker to evaluate disease activity in patients with ulcerative colitis.	100%
Statement 4	Fecal calprotectin levels are correlated with endoscopic and histological disease activity in patients with ulcerative colitis.	<75% *
Statement 5	A fecal calprotectin value greater than 250 µg/g is associated with disease activity.	<75% *
Statement 6	Fecal calprotectin is a reliable biomarker to evaluate the response to treatment and to monitor disease activity in patients with ulcerative colitis.	<75% *
Statement 7	Fecal calprotectin is a predictor of clinical disease relapse and clinical remission.	<75% *
Statement 8	Normalization of fecal calprotectin values is a treatment target in patients with ulcerative colitis.	73%
Statement 9	Escalation therapy should be performed in case of elevated fecal calprotectin values and persistent clinical activity of disease.	<75% *
Statement 10	Fecal calprotectin should be measured before starting any therapy for ulcerative colitis.	100%
Statement 11	Fecal calprotectin should be measured at the end of induction therapy and then every 3–4 months during the maintenance phase.	<75% *
Statement 12	Fecal calprotectin should be measured in case of clinical exacerbation of disease.	<75% *
Statement 13	Home measurement of fecal calprotectin is a reliable method for monitoring disease activity in patients with mild-to-moderate ulcerative colitis.	<75% *

* During the preliminary discussion of the statement, agreement was not reached, and it was decided to directly amend the statement without a formal vote.

**Table 2:** Approved statements regarding the measurement of fecal calprotectin in the management of patients with inflammatory bowel diseases.

Statement 1	Fecal calprotectin is a reliable biomarker for differentiating ulcerative colitis from functional disorders.	(agreement 100%)
Statement 2	Fecal calprotectin is a reliable biomarker to evaluate disease activity in patients with ulcerative colitis.	(agreement 100%)
Statement 3	Fecal calprotectin levels are correlated with endoscopic disease activity in patients with ulcerative colitis.	(agreement 100%)
Statement 4	Fecal calprotectin levels are correlated with histological disease activity in patients with ulcerative colitis.	(agreement 92%)
Statement 5	A fecal calprotectin value greater than 250 µg/g suggests the presence of active disease.	(agreement 100%)
Statement 6	Fecal calprotectin is a reliable biomarker to evaluate the response to treatment in patients with ulcerative colitis.	(agreement 100%)
Statement 7	Fecal calprotectin is a reliable biomarker to monitor disease activity in patients with ulcerative colitis.	(agreement 100%)
Statement 8	Persistent high values of fecal calprotectin predict clinical disease relapse in asymptomatic patients.	(agreement 100%)
Statement 9	Fecal calprotectin should be a treatment target in patients with ulcerative colitis.	(agreement 92%)
Statement 10	Fecal calprotectin should be measured before starting any therapy for ulcerative colitis.	(agreement 100%)
Statement 11	Fecal calprotectin should be measured before optimizing any therapy for ulcerative colitis.	(agreement 100%)
Statement 12	Fecal calprotectin should be measured at the end of induction therapy, during maintenance, or in case of clinical exacerbation of disease.	(agreement 100%)
Statement 13	Escalation therapy should be performed in asymptomatic patients in case of persistent elevated fecal calprotectin values.	(agreement 83%)
Statement 14	Home measurement of fecal calprotectin is a useful and reliable method for monitoring disease activity in patients with ulcerative colitis.	(agreement 100%)

## Data Availability

No new data were generated or analyzed in support of this research.

## Supplementary Materials

The following supporting information can be downloaded at: https://www.mdpi.com/article/10.3390/jcm12031142/s1, Supplementary Table S1: Studies reporting data on home measurement of fecal calprotectin and included in the systematic review; Supplementary Figure S1: Flow chart of the screening strategy to identify the studies.
