# Supplementary material for: iSTART-II: An Update on the i Support Therapy–Access to Rapid Treatment (iSTART) Approach for Patient-Centered Therapy in Mild-to-Moderate Ulcerative Colitis
Source: J Clin Med. 2023 Feb 1;12(3):1142. doi: 10.3390/jcm12031142 (PMC9918267; doi:10.3390/jcm12031142)

Supplementary Table S1: Studies reporting data on home measurement of fecal calprotectin and included in the systematic review

First author	Publication Year	Study design	Study population	n of patients	Study arms	Outcome measure	Main results	Reading error rate	Usability
Haisma [43]	2019	Prospective cohort study	IBD	1560 stool samples	IBDoc<br>QuantOnCal<br>CalproSmart<br>BUHLMANN fCAL<br>IDK Calprotectin<br>Calprotectin (ALP)	Agreement between the home test and the companion ELISA	**FC ≤ 500 µg/g**<br>IBDoc 87%<br>QuantOn Cal 82%<br>CalproSmart 76%<br>**FC ≥ 500 µg/g**<br>IBDoc 37%<br>QuantOn Cal 19%<br>CalproSmart 37%	IBDoc 1.9%<br>QuantOn Cal 4.8%<br>CalproSmart 5.8%	NA
Elkjaer [20]	2010	Post-hoc analysis of randomized controlled trial	UC	404 stool samples	Home test<br>Rapid test<br>ELISA test	Assay reproducibility	Home test 24.5%<br>Rapid test 21.1%<br>ELISA 19.8%	NA	NA
Heida [44]	2017	Prospective cohort study	IBD	101 patients, 460 stool samples	IBDoc<br>QuantumBlue<br>ELISA test	Agreement between IBDoc, QuantumBlue and ELISA	**FC ≤ 500 µg/g**<br>IBDoc-QuantumBlue 91%<br>IBDoc-ELISA 71%<br>**FC ≥ 500 µg/g**<br>IBDoc-QuantumBlue 81%<br>IBDoc-ELISA 64%	NA	87% not difficult to perform
Ankersen [45]	2019	Randomized clinical trial	IBD	102 patitents	CalproSmart every 3 months<br>CalproSmart on demand	Difference in clinical activity, adherence to therapy, and fatigue	No difference was found	NA	NA
Ankersen [46]	2020	Prospective cohort study	IBD	78 patients, 288 stool samples	CalproSmart	Associations between faecal microbiota and disease activity	Peptostreptococcus anaerobius was found to correlate significantly with increasing FC	NA	NA
Vinding [47]	2016	Randomized clinical trial	IBD	221 patients, 1526 stool	CalproSmart<br>ELISA test	Variability, accuracy, and comparison with ELISA	intra-assay CV=4.42%<br>interassay CV=12.49%<br>sensitivity*= 89%<br>specificity*=76%	NA	NA

				samples			PPV*=53%<br>NPV*=96%<br>r=0.685 p< 0.0001		
Bello [48]	2017	Prospective cohort study	IBD	58 patients	IBDoc<br>ELISA test	Adherence to measurement and usability	46.6% adherence<br>GSU 74 at day 0	NA	GSU 74 at day 0
Ashorov [49]	2020	Prospective cohort study	IBD/IBS	100 stool samples	Liaison XL<br>QuantOn Cal	Correlation between the 2 tests	Sensitivity=98.7%<br>Specificity=76.2%<br>Accuracy=94.0%<br>r = 0.82, p < 0.0001	NA	NA
Moore [50]	2019	Prospective cohort study	IBD	61 patients	IBDoc<br>ELISA test	Usability and accuracy of IBDoc	easy to use=79%<br>Pearson correlation coefficient=0.88	NA	easy to use=79%
Wei [51]	2018	Prospective cohort study	IBD	51 patients, 68 stool samples	Quantum Blue<br>IBDoc	Correlation between the 2 tests	r=0.776, P<0.0001<br>agreement= 80% (FC cutoff=250 μg/g)	NA	easy to use=56%
Puolanne [52]	2019	Randomized clinical trial	IBD	123 patients, 301 stool samples	CalDetect<br>ELISA test	Feasibility of a self-monitoring strategy	r=0.740, p< 0.05<br>decreased rate of outpatient visits (24% vs 40%, p<0.05)<br>adherence at 12 months (20% vs 38%, p<0.001)	NA	simple: 80%
D'Amico [19]	2020	Prospective cohort study	IBD	20 patients	IBDoc	Usability	easy to use=95%<br>patients'satisfaction=85%	NA	easy to use=95%
Östlund [60]	2021	Randomized clinical trial	IBD	158 patients	IBDoc	Compliance	Compliance=29%<br>Increased medical treatment (33% vs 15%, p<0.007)	NA	easy to use=82%
Orfanoudaki [53]	2021	Retrospective cohort study	IBD	72 patients	IBDoc	Compliance	Compliance=90.3%<br>FC correlated with endoscopically active disease (OR: 1.003; 95%CI, 1.001-1.006, p<0.01)<br>AUC=0.78[§]	NA	NA
Jere [54]	2021	Prospective cohort study	IBD	54 pediatric patients	IBDoc	Feasibility of a self-monitoring strategy	Compliance=76%<br>IBDoc based treatment changes= 31.7%	NA	easy to use=70%
Lerchova	2019	Prospective	IBD	89	IBDoc	To compare the	AUC for IBDoc= 0.792	NA	NA

[55]		cohort study		pediatric patients, 102 stool samples	ELISA test	accuracy IBDoc and ELISA<br>To compare the variability of the results	(optimal cut-off =48mg/g)<br>ELISA was more accurate than IBDoc (p<0.023)<br>ICC=0.79, 95%CI 0.53–0.89		
de Jong [56]	2019	Prospective cohort study	IBD	109 patients	QuantOn Cal<br>CalproSmart<br>ELISA test	Accuracy	QuantOn Cal (cut-off=250 μg/g)<br>sensitivity=82.4%<br>specificity=87.1%<br>AUC=0.84<br>r=0.75 (p<0.001)<br>CalproSmart (cut-off=100 μg/g)<br>sensitivity=76.9%<br>specificity=58.3%<br>AUC=0.75<br>r=0.89 (p<0.001)	NA	NA
Piekkala [57]	2018	Prospective cohort study	IBD	35 pediatric patients, 197 stool samples	IBDoc<br>ELISA test	Feasibility of the FC home monitoring	30 tests (15%) failed<br>r=0.785; p < 0.0001	NA	equal to or better than ELISA=47%
McComb ie [58]	2020	Randomized clinical trial	IBD	100 patients	IBDoc<br>ELISA test	Non-inferiority in quality of life compared with standard	No difference in IBDQ at 12 months (181.1 vs 170.6, p>0.05)<br>Adherence to IBDoc=30%	NA	Mean SUS=71.6
Hejl [59]	2017	Prospective cohort study	IBD	55 stool samples	IBDoc<br>ELISA test	Correlation	r=0.887<br>CVs at 3 different FC levels were in the range of 4.8–26.6%	NA	NA
Parr [60]	2016	Prospective cohort study	IBD	54 patients	IBDoc<br>ELISA test	Adherence	Adherence: 35%<br>r = 0.77, p < 0.0001	NA	Preference for IBDoc=85%

FC: fecal calprotectin; IBD: inflammatory bowel diseases; UC: ulcerative colitis; n: number; CV: coefficient of variation; PPV: positive predictive value; NPV: negative predictive value; GSU: Global Score of Usability; IBS: irritable bowel syndrome; OR: odds ratio; CI: confidence interval; AUC: area under the curve; ICC: Intraclass correlation coefficient; SUS: System usability scale;

* FC ≥ 200 mg/g to predict disease activity

§ FC identified patients with endoscopically active disease more effectively than other biomarkers

Search strategy

The Cochrane Handbook and the Preferred Reporting Items for Systematic Reviews and MetaAnalyses (PRISMA) statement for reporting of systematic reviews were used as guidance for this systematic review. We searched in Pubmed, Cochrane library, and Web of Science databases to identify all studies reporting measurement of FC at home in patients with IBD up to May 2022. Studies evaluating non-IBD patients or those in which fecal calprotectin was not measured at home were excluded. Furthermore, reviews, editorials, commentaries, and letters were also excluded. The following Medical Subject Heading (MeSH) terms alone or matched with the Boolean operators ‘AND’ or ‘OR’ were used: “ulcerative colitis”, “UC”, “Inflammatory Bowel Disease”, “IBD”, “mild to moderate”, “fecal calprotectin”, “home measurement”, “home”, “monitoring”. Titles and abstracts were independently evaluated by two reviewers (FD and BC) to identify eligible studies. Then, full-text articles were examined for inclusion. In addition, reference lists of included articles were reviewed to identify any missing papers. In case of disagreement, a third author was involved to resolve any doubts.

Supplementary Figure S1: Flow chart of the screening strategy to identify the studies

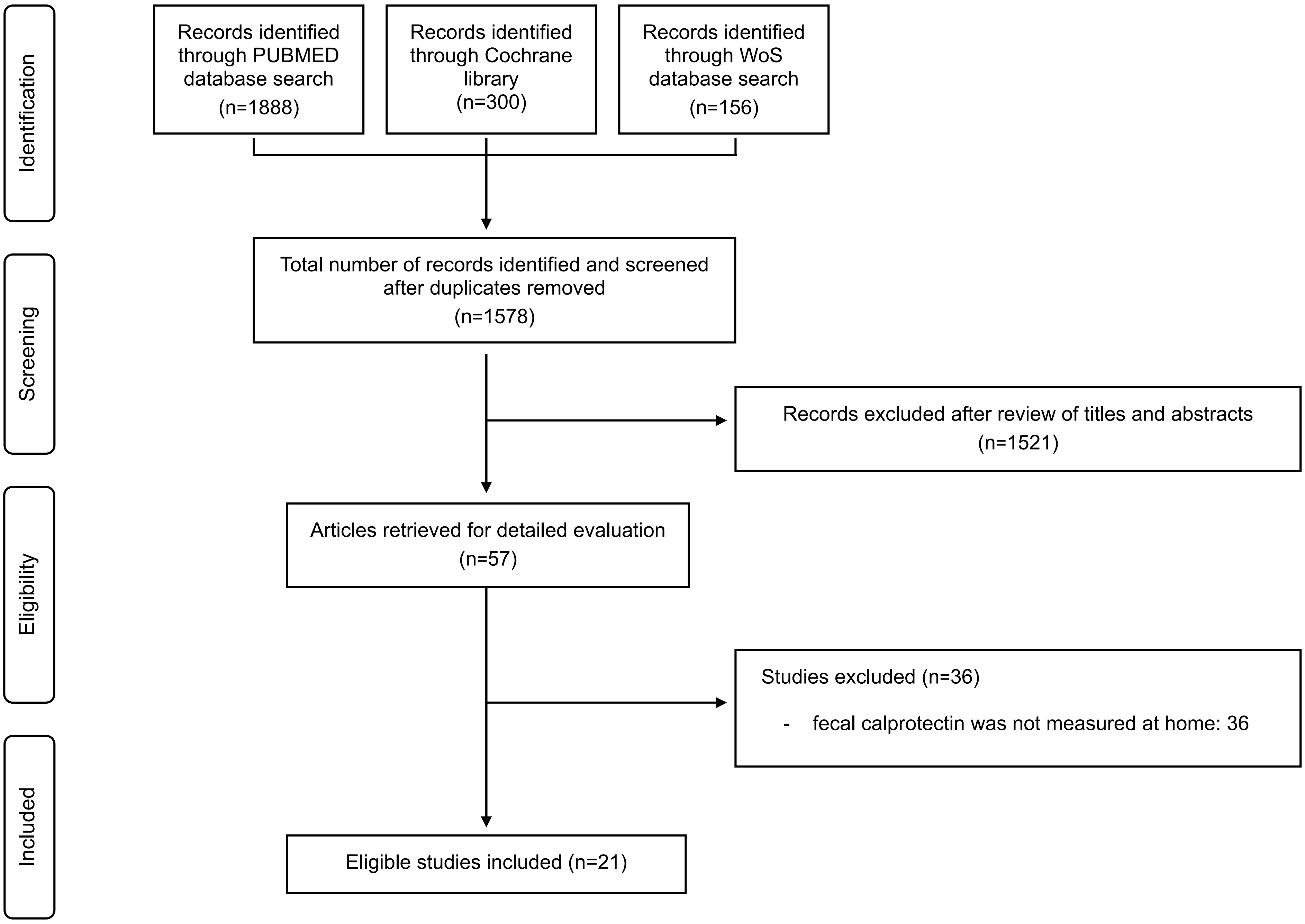

Supplement: Supplementary file 1 [file jcm-12-01142-s001.zip › jcm-2118030-SI.pdf]
